# Ciliate *Paramecium* is a natural reservoir of *Legionella pneumophila*

**DOI:** 10.1038/srep24322

**Published:** 2016-04-15

**Authors:** Kenta Watanabe, Ryo Nakao, Masahiro Fujishima, Masato Tachibana, Takashi Shimizu, Masahisa Watarai

**Affiliations:** 1 The United Graduate School of Veterinary Science, Yamaguchi University, Yamaguchi, Japan; 2 Joint Faculty of Veterinary Medicine, Laboratory of Veterinary Public Health, Yamaguchi University, Yamaguchi, Japan; 3 The Graduate School of Veterinary Medicine, Hokkaido University, Sapporo, Japan; 4 Department of Environmental Science and Engineering, Graduate School of Science and Engineering, Yamaguchi University, Yamaguchi, Japan; 5 National BioResource Project of Japan Agency for Medical Research and Development, Chiyoda-ku, Tokyo, Japan; 6 Division of Biomedical Food Research, National Institute of Health Sciences, Setagaya-ku, Tokyo, Japan

## Abstract

*Legionella pneumophila*, the causative agent of Legionnaires’ disease, replicates within alveolar macrophages and free-living amoebae. However, the lifestyle of *L. pneumophila* in the environment remains largely unknown. Here we established a novel natural host model of *L. pneumophila* endosymbiosis using the ciliate *Paramecium caudatum*. We also identified *Legionella* endosymbiosis-modulating factor A (LefA), which contributes to the change in life stage from endosymbiosis to host lysis, enabling escape to the environment. We isolated *L. pneumophila* strains from the environment, and they exhibited cytotoxicity toward *P. caudatum* and induced host lysis. Acidification of the *Legionella*-containing vacuole (LCV) was inhibited, and enlarged LCVs including numerous bacteria were observed in *P. caudatum* infected with *L. pneumophila*. An isogenic *L. pneumophila lefA* mutant exhibited decreased cytotoxicity toward *P. caudatum* and impaired the modification of LCVs, resulting in the establishment of endosymbiosis between them. Our results suggest that *L. pneumophila* may have a mechanism to switch their endosymbiosis in protistan hosts in the environment.

Symbiotic relationships between different species are thought to have a huge impact on the dynamics of organic evolution and biotic diversity. Bacteria in the natural environment are able to benefit by establishing these symbiotic relationships with protistan hosts^1^. It has been reported that some obligate intracellular bacteria, such as *Neochlamydia, Parachlamydia,* and *Rickettsia,* adapt to the intracellular conditions in free-living amoebae and establish an endosymbiotic relationship^2^,^3^,^4^. It could be speculated that these bacteria selected the lifestyle of a symbiont during the course of evolution because it provides advantages. Protistan hosts can protect the symbionts from various environmental stresses and provide an ideal environment for bacterial replication^5^,^6^. These benefits are thought to be advantageous for the survival of bacterial symbionts. Furthermore, previous studies have demonstrated that the evolution of bacterial pathogenesis and the acquisition of virulence toward humans and/or animals correlate with the virulence toward the protistan host^7^,^8^,^9^,^10^. It has also been indicated that newly isolated amoebae-resistant bacteria are pathogenic for humans and/or animals^11^,^12^.

*Legionella pneumophila,* the major causative agent of Legionnaires’ disease and Pontiac fever^13^,^14^, is a facultative intracellular Gram-negative bacterium that can establish an endoplasmic reticulum-derived vacuole, in which it replicates, by utilizing the *dot/icm* genes, which encode a type IVB secretion system (T4SS)^15^,^16^,^17^,^18^. This T4SS makes it possible for *L. pneumophila* to replicate in human alveolar macrophages. However, human infection is a dead end for *L. pneumophila,* from an evolutionary point of view, because human-to-human spread of *L. pneumophila* has not been observed^19^,^20^.

*L. pneumophila* is often found in natural and man-made aquatic environments where it can survive for long periods^21^,^22^. From a public health perspective, it can be a high-impact risk factor for human infection. Furthermore, it is well known that *L. pneumophila* can also survive in the environment in close association with free-living protists. Amoebae and *Tetrahymena* have been reported to be protistan hosts of *Legionella* in environmental fresh water^23^,^24^. This association leads to the replication and spread of *L. pneumophila* in the environment and the development of antibiotic resistance^25^. In addition, protistan hosts are thought to be the primary evolutionary factors for the acquisition and maintenance of virulence toward humans^20^,^26^,^27^. Although previous reports have suggested that the Dot/Icm system also contributes to intracellular survival and replication in amoeba^20^,^28^, the mechanisms of infection and endosymbiosis in protistan hosts are not fully understood.

So far, 14 species of amoebae and 2 species of ciliates are known as potential hosts of *L. pneumophila*^23^,^29^. *Paramecium* spp., found widely in environmental water, are free-living, single-celled, freshwater ciliates that feed on bacteria^30^. *Paramecium* spp. are appreciated as model organisms not only for the analysis of cellular and molecular biology, including phagocytosis and exocytosis^31^,^32^, but also for endosymbiosis^33^,^34^,^35^. *Holospora* spp. are endonuclear symbionts of the *Paramecium* spp^36^. *P. caudatum*, for example, can acquire salinity resistance^37^ and heat-shock resistance^38^,^39^,^40^ if infected with *Holospora* spp. Despite the fact that many endosymbiotic bacteria have been identified in *Paramecium* spp.^41^, interactions between *Paramecium* spp. and *Legionella* spp. are unknown.

In this study, we generated a novel protistan host for *L. pneumophila* and investigated the mechanisms by which *L. pneumophila* establishes endosymbiosis in the model. We observed not only the process of endosymbiosis but also a cytotoxicity that rejects endosymbiosis with *L. pneumophila* strains from *Paramecium* hosts. Our results suggest that *L. pneumophila* has functional systems to control endosymbiosis with *Paramecium* spp., including the selection of suitable host strains in the environment.

## Results

### *Paramecium* spp. as a novel host model of *Legionella* endosymbiosis

**Figure 1:**
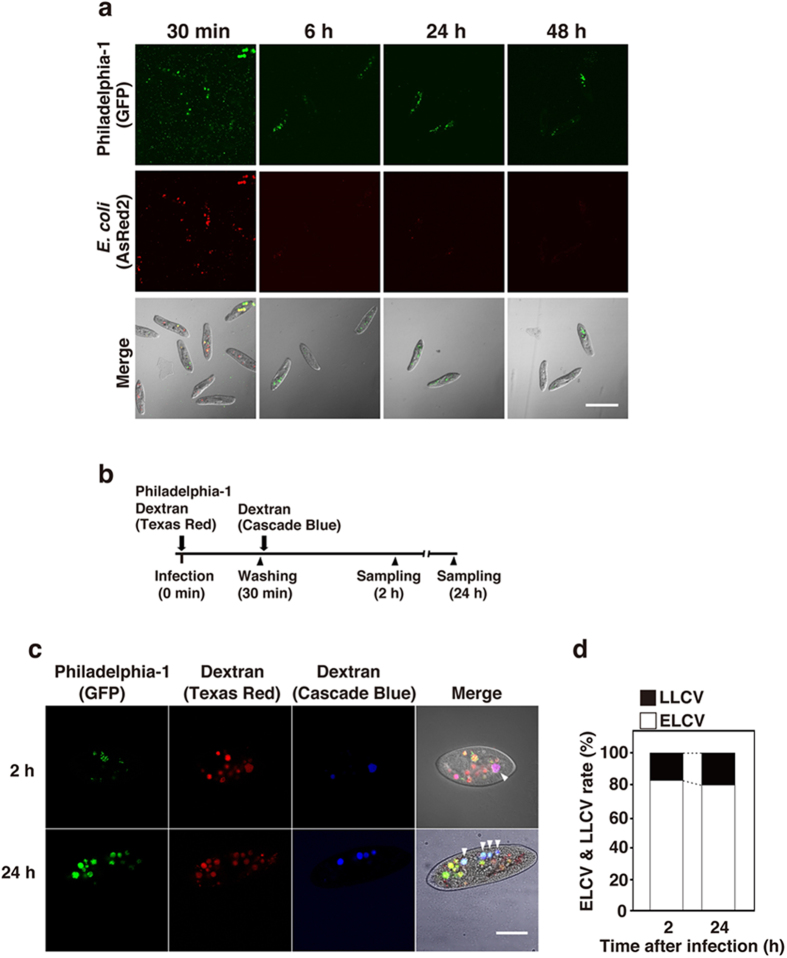
*L. pneumophila* Philadelphia-1 establishes endosymbiosis in *P. caudatum* RB-1. (**a**) Intracellular localization of Philadelphia-1 (GFP) and *E. coli* (AsRed2) in RB-1 at 30 min, 6 h, 24 h, and 48 h after infection. Bacteria were simultaneously added to RB-1 at an MOI of 10000. Scale bar represents 100 μm. (**b**) Bacteria and each dextran were added to RB-1 according to this schedule. LCVs containing each dextran were observed by confocal laser scanning microscopy 2 h and 24 h after infection (**c**), and percentages of ELCV and LLCV are shown with the total of all LCVs being 100% (**d**). Arrowheads point to LLCV, which are LCVs positive for both Texas Red- and Cascade Blue-conjugated dextrans. Scale bar represents 30 μm.

We established a novel host model of *Legionella* symbiosis using *Paramecium* spp. *P. caudatum* strain RB-1 was infected with *L. pneumophila* strain Philadelphia-1 (Phi-1) by mixing them; then the intracellular localization of the bacteria was determined. *Escherichia coli* used as a control were observed inside the phagosomes of *P. caudatum* RB-1 30 min after infection. *L. pneumophila* Phi-1 remained in *P. caudatum* RB-1 without digestion for at least 48 h after infection (Fig. 1a). In contrast, *E. coli* were reduced in number 6 h after infection, and disappeared completely within 48 h after infection (Fig. 1a). It is well known that *P. caudatum* have high phagocytic and exocytic activities^42^. Hence, it is possible that re-phagocytosis of free *Legionella* released from other *P. caudatum* RB-1 by exocytosis occurs in this assay. To confirm this possibility, a fluorescent dye-conjugated dextran uptake assay was performed according to the schedule shown in Fig. 1b. The percentage of early-formed *Legionella*-containing vacuoles (ELCV), which are only Texas Red-conjugated dextran (TRDx) positive, and that of late-formed *Legionella*-containing vacuoles (LLCV), which are positive for both TRDx and Cascade Blue-conjugated dextran (CBDx), were measured 2 and 24 h after infection. As a result, the rate of ELCV (82.7%) was higher than that of LLCV (17.3%) 2 h after infection (Fig. 1c,d). The ELCV were so stable in RB-1, such that the distribution between ELCV and LLCV was hardly changed 24 h after infection (ELCV, 80%; LLCV, 20%) (Fig. 1c,d). These results suggest that *P. caudatum* RB-1 is likely to serve as a protistan host of *Legionella. Legionella* may establish endosymbiosis and survive in *Paramecium* by controlling the turnover of LCV.

### Cytotoxicity toward *Paramecium* spp. infected with *Legionella*

To assess whether endosymbiosis is an inducible phenomenon only between *L. pneumophila* Phi-1 and *P. caudatum* RB-1, 62 strains of *Paramecium* spp. and 8 strains of *L. pneumophila* were examined, in all combinations, using the same infection assay. Although most *L. pneumophila* strains, particularly the 3 strains isolated from human lung, had no effect on host viability, only 2 strains, Ofk308 and Bnt314, isolated from environmental water exhibited cytotoxicity toward 8 and 15 strains of *Paramecium* within 48 h after infection, respectively (Table 1). The *P. caudatum* RB-1 strain used in Fig. 1 was also sensitive to their cytotoxicity. Thus, *P. caudatum* RB-1 was employed as a model host strain in the following experiments.

**Figure 2:**
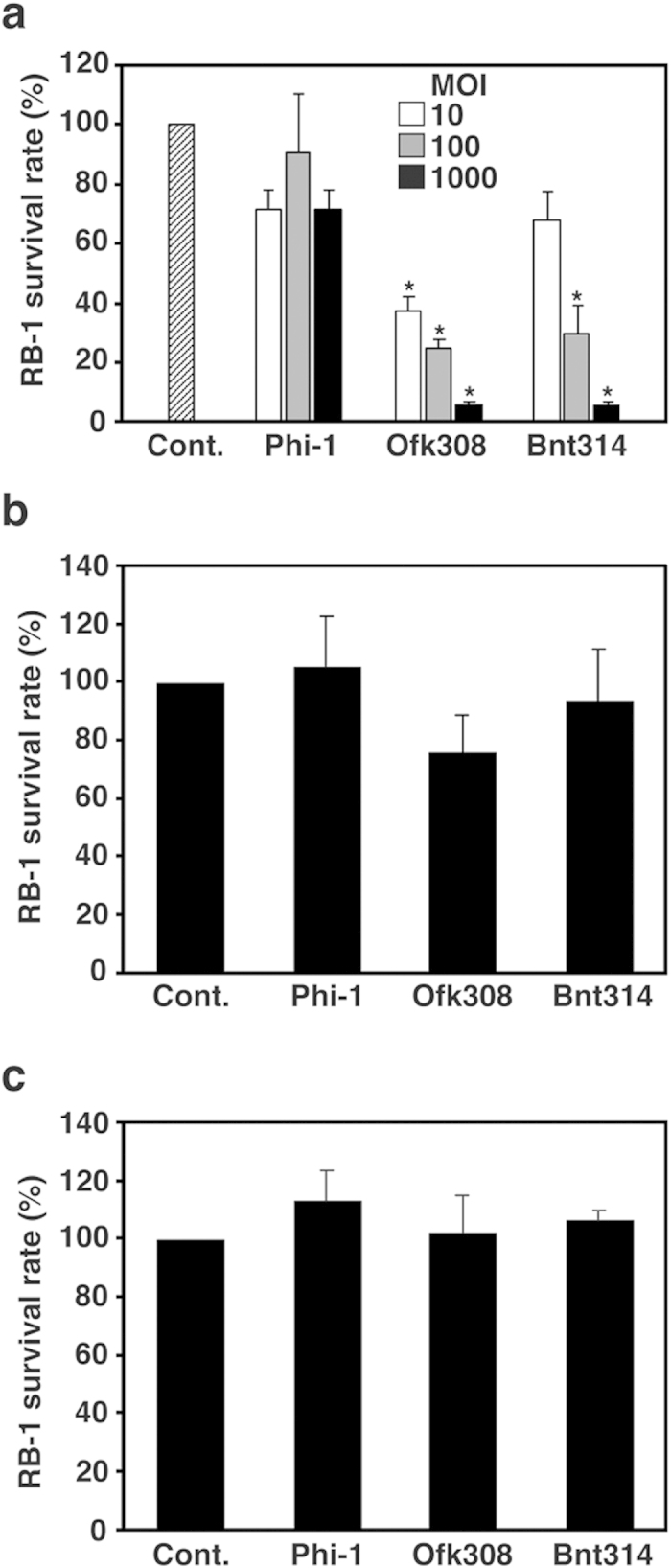
Infection of *L. pneumophila* Ofk308 is required to exhibit the cytotoxicity toward RB-1. (**a**) RB-1 was infected with *L. pneumophila* Phi-1, Ofk308, and Bnt314 at MOIs of 10, 100, and 1000. RB-1 was treated with culture supernatants of Phi-1, Ofk308, and Bnt314 (**b**), or with killed bacteria (**c**). Cont., no infection. Relative RB-1 survival rates are indicated, with Cont. being defined as 100%. Data are averages of triplicate samples from three identical experiments, and error bars represent standard deviations. Statistically significant differences, compared with Cont., are indicated by asterisks (*P < 0.01).

In order to investigate the mechanisms of cytotoxicity, *P. caudatum* RB-1 was infected with *L. pneumophila* Ofk308 and Bnt314 for 48 h at various multiplicities of infection (MOIs). In this experiment, Ofk308 and Bnt314 infection clearly caused cytotoxicity in an MOI-dependent matter (Fig. 2a). Next, *P. caudatum* were treated with culture supernatants of these two strains of bacteria for 48 h. This treatment did not affect the number of live *P. caudatum* (Fig. 2b). When *P. caudatum* was mixed with killed bacteria, no difference was observed from the non-fed control (Fig. 2c). These results indicate that active live bacterial uptake to the host phagosomes is required to bring out their cytotoxicity. Most *Legionella* strains have the ability to establish endosymbiosis with *Paramecium.* However, environmental isolates of *L. pneumophila*, such as Ofk308 and Bnt314, do not have this ability, due to unknown factors that are cytotoxic for host cells at environmental temperatures.

### Isolation of a cytotoxicity-defective mutant of *L. pneumophila* Ofk308

**Figure 3:**
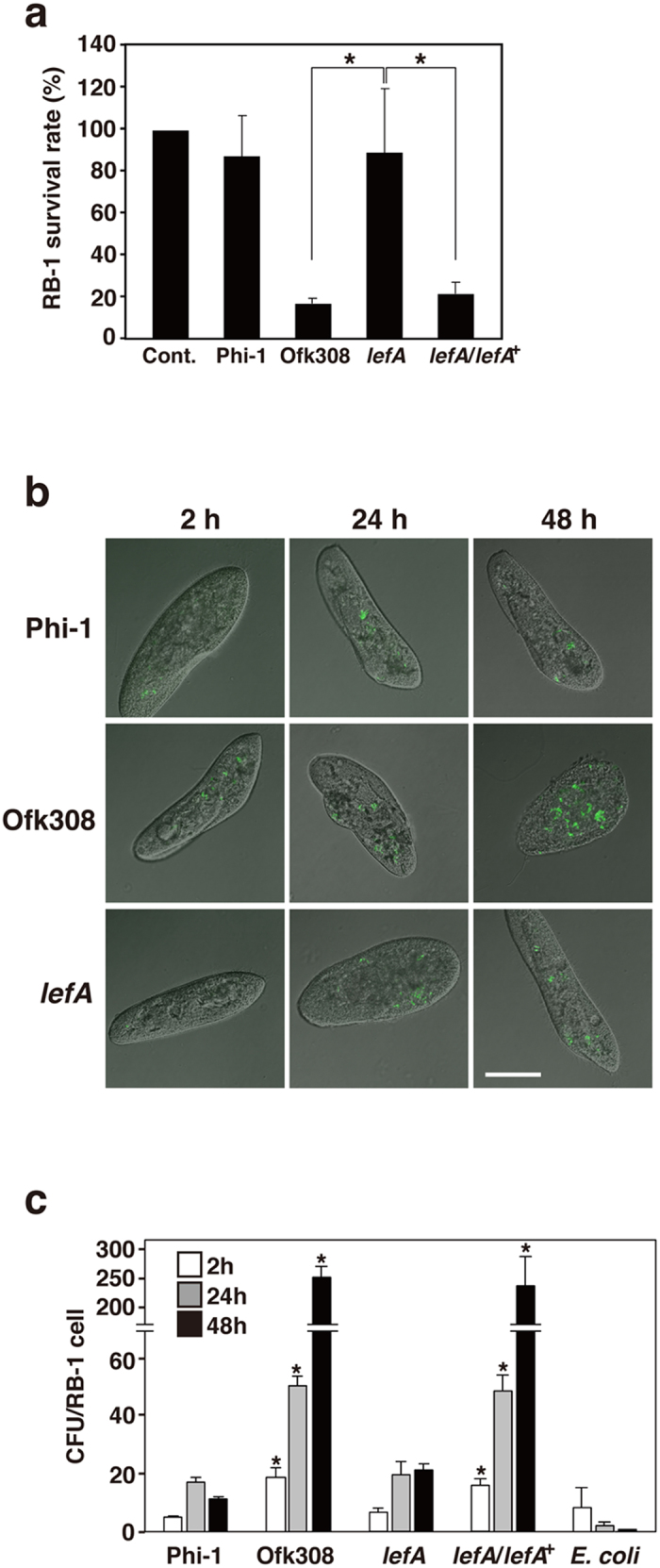
*lefA* mutant has no cytotoxicity toward RB-1. (**a**) RB-1 was infected with Phi-1, Ofk308, the *lefA* mutant (*lefA*), and the *lefA* complemental strain (*lefA*/*lefA*^+^) at MOIs of 1000. Cont., no infection. Relative RB-1 survival rates are indicated, with Cont. being defined as 100%. (**b**) Cell shapes of RB-1 infected with each strain of *L. pneumophila* 2 h, 24 h, and 48 h after infection. Scale bar represents 30 μm. (**c**) Number of bacteria per RB-1 cell. Data are averages of triplicate samples from three identical experiments, and error bars represent standard deviations. Statistically significant differences compared with *lefA* (**a**) or Phi-1 (**c**) are indicated by asterisks (*P < 0.01).

To identify the factors contributing its cytotoxicity toward *P. caudatum* RB-1, we mutagenized *L. pneumophila* Ofk308 randomly with the mini-Tn5Km2 transposon. Mutants carrying the transposon inserted into the chromosome were resistant to kanamycin, and were selected on BCYE agar plates containing kanamycin. Using this method, 240 transconjugants were isolated and then screened in a *P. caudatum* infection assay. Finally, 4 cytotoxicity-defective mutants were isolated. We tried to identify the transposon insertion site in these 4 mutants, but 3 of them were not identified. We identified the mutated gene in only one mutant. The gene was lpofk01540, which is a homolog of the sodium/hydrogen antiporter of *L. pneumophila* Phi-1 (lpg1507). It is likely that the gene is involved in establishing endosymbiosis in *Paramecium*. Therefore, we named the gene “*Legionella* endosymbiosis-modulating factor A (*lefA*).” This *lefA* mutant has lost its cytotoxicity toward *P. caudatum* to the same level as *L. pneumophila* Phi-1, and its complemental strain showed recovery of cytotoxicity comparable with that of the parental strain Ofk308 (Fig. 3a). We also evaluated the intracellular growth of bacterial strains in *P. caudatum* LCVs. *L. pneumophila* Phi-1 and *lefA* mutant showed slight growth in *P. caudatum* and no damage was observed in infected *P. caudatum* (Fig. 3b,c). The number of Ofk308 increased significantly in *P. caudatum* and the shapes of infected *P. caudatum* were changed unnaturally (Fig. 3b,c), which is likely to lead to cell death due to eventual rupture. The *lefA* complemental strain also showed high intracellular growth efficiency and damaged infected *P. caudatum. E. coli* was digested and its numbers reduced rapidly, as shown in Figs 1a and 3c.

### The expression of *lefA* in *L. pneumophila* Ofk308

**Figure 4:**
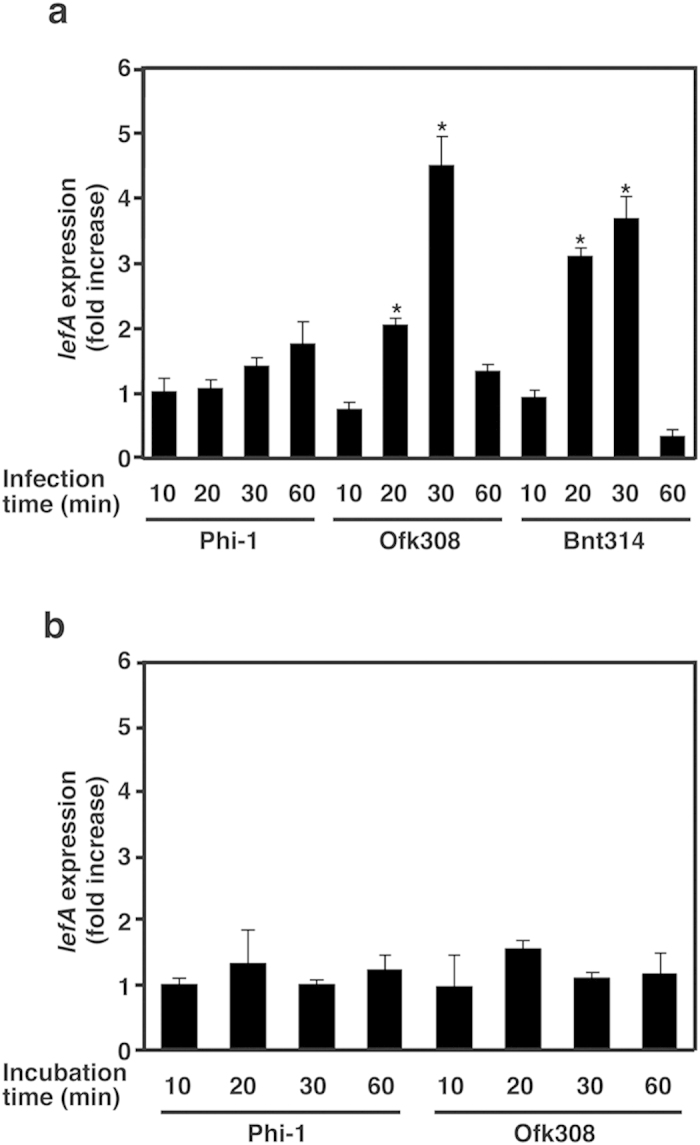
Expression of *lefA* in Ofk308 and Bnt314 is upregulated within RB-1. (**a**) Each strain of *L. pneumophila* was used to infect RB-1 for 10 to 60 min, and RNA samples were collected from the bacteria. Expression of *lefA* was determined by real-time PCR. The fold increase of *lefA* was normalized to 16S rRNA; the expression levels are represented relative to a sample obtained 10 min after infection with Phi-1. (**b**) Each strain was incubated at 25 °C for 10 to 60 min without RB-1, and then RNA samples were collected. The expression was determined as described above. Statistically significant differences compared to Phi-1 are indicated by asterisks (*P < 0.01).

Although the results described above suggest that *lefA* contributes to the cytotoxicity toward *P. caudatum*, all *Legionella* strains used in this study have a homolog of *lefA,* regardless of their cytotoxicity. Because the LefA protein of Ofk308 shares 99.2% amino acid sequence homology with that of Phi-1 and these sequence analyses did not clearly explain the relationship between amino acid profile and cytotoxicity (Supplementary Fig. 1), we determined the expression levels of these genes using quantitative real time PCR. The expression of Ofk308 *lefA* was significantly increased within *P. caudatum* from 20 to 30 min after infection (Fig. 4a); although, it was stable under *in vitro* culture conditions and no significant difference was observed when compared with the Phi-1 *lefA* (Fig. 4b). Expression of the *lefA* in Bnt314, which has the same cytotoxicity as Ofk308 (Fig. 2a), was also upregulated by infection. On the other hand, such inducible upregulation of *lefA* was not observed in Phi-1.

### *L. pneumophila* Ofk308 modulates LCV acidification in RB-1

**Figure 5:**
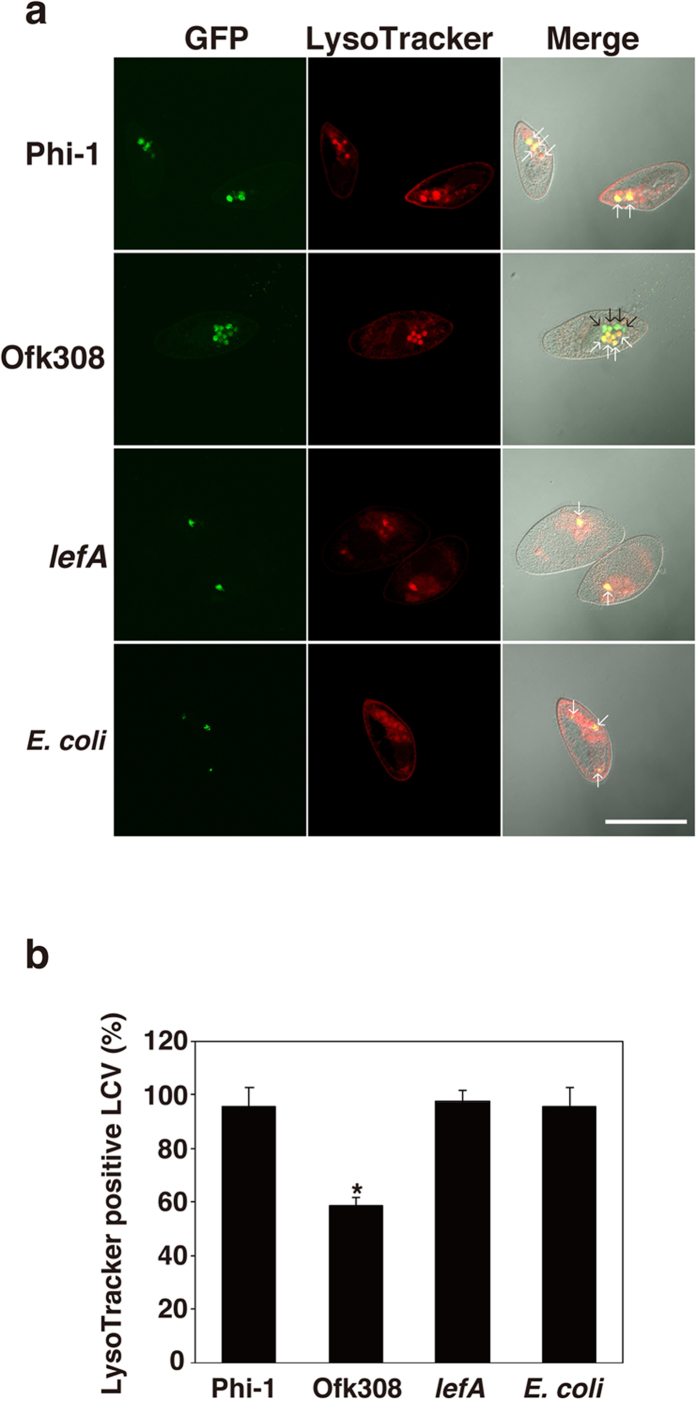
The maturation of host LCVs containing Ofk308 is inhibited. (**a**) LCV maturation 30 min after infection was evaluated with LysoTracker. Bacteria were added to RB-1 at an MOI of 10000. White arrows point to LysoTracker-positive LCVs (or phagosomes containing *E. coli*). Black arrows point LysoTracker-negative LCVs. Scale bar represents 100 μm. (**b**) Relative LysoTracker-positive LCVs percentages are shown, with the total of all LCVs being 100%. Data are the averages of triplicate samples from three identical experiments, and error bars represent standard deviations. Statistically significant differences compared to Phi-1 are indicated by asterisks (*P < 0.01).

To investigate how *lefA* modulates the endosymbiosis of Ofk308 in *Paramecium*, the maturation process of *P. caudatum* phagosomes that contain bacteria was evaluated using a LysoTracker. As a result, almost all of the phagosomes containing *L. pneumophila* Phi-1 (95.8%), the *lefA* mutant (97.4%), or *E. coli* (95.8%) were LysoTracker-positive 30 min after infection (Fig. 5a,b), indicating that majority of these phagosomes are acidified. In contrast, the percentage of the LysoTracker-positive phagosomes containing Ofk308 was lower (58.9%) (Fig. 5a,b).

**Figure 6:**
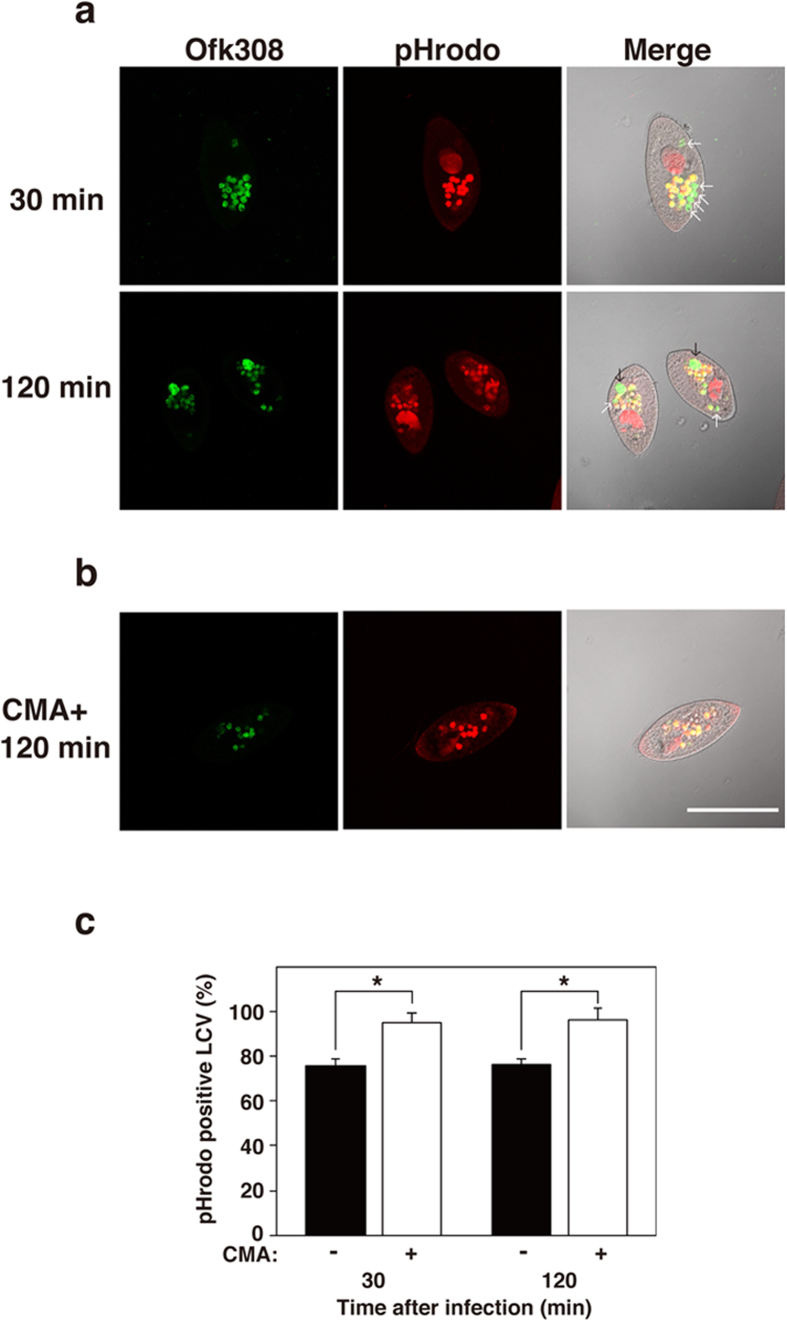
Concanamycin A treatment affects the acidification and enlargement of host LCVs formed by infection of Ofk308. RB-1 was infected with Ofk308 at an MOI of 10000 and pHrodo-conjugated dextran was added simultaneously (**a**) without or (**b**) with concanamycin A (CMA). White arrows point to pHrodo-negative LCVs. Black arrows point to enlarged LCVs. Scale bar represents 100 μm. (**c**) Relative pHrodo-positive LCVs percentages are shown, with the total of all LCVs being 100%. Data are the averages of triplicate samples from three identical experiments, and error bars represent standard deviations. Statistically significant differences are indicated by asterisks (*P < 0.01).

To address the acidification of phagosomes containing Ofk308 in detail, pH indicator (pHrodo)-conjugated dextran (pHDx) and Ofk308 were added to a *P. caudatum* culture at the same time. Thirty min after infection, 76% of the LCVs exhibited low pH and 24% of the LCVs were negative for pHDx, as in the experiment with LysoTracker shown in Figs 5 and 6a,c. Furthermore, one or two enlarged LCVs per host cell were also observed 2 h after infection. The enlarged LCVs are a quite unique structure observed in *P. caudatum* infected with Ofk308 at high MOI (Fig. 6a). These enlarged LCVs were also completely negative for pHDx, which means that their acidification was inhibited. These results suggest that Ofk308 modifies both the size and pH of LCVs.

Concanamycin A (CMA) is a specific inhibitor of vacuole-type ATPase (V-ATPase). It has been reported that the CMA inhibits the acidification of *P. caudatum* phagosomes^43^. We investigated the effects of CMA treatment on LCV modification by Ofk308 infection in *P. caudatum*. When *P. caudatum* were pre-treated with CMA before Ofk308 infection, these treatments reduced the phagocytosis of *P. caudatum*, which made it difficult to observe enough LCVs (Supplementary Fig. 2). Thus, we treated *P. caudatum* with CMA 5 min after infection with Ofk308. CMA treatment kept 95% of the LCVs in a low pH condition from 30 min to 2 h (Fig. 6b,c). Moreover, the enlarged LCVs did not appear at all. We have confirmed that CMA treatment had no direct effect on Ofk308 viability itself at the concentration used in this assay (Supplementary Fig. 3). These results suggest that the modulation of LCVs by Ofk308 depends on *P. caudatum* V-ATPase.

### Intracellular growth of *lefA* mutant in THP-1 cells

**Figure 7:**
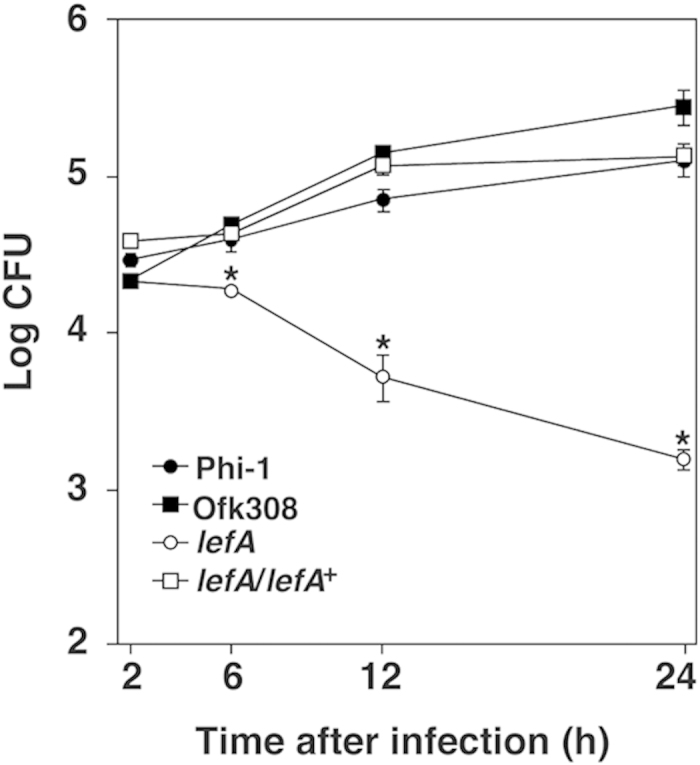
*lefA* mutant fails to grow in human monocytic THP-1 cells. Infected THP-1 cells were cultured for 2, 6, 12, and 24 h. Data are the averages of triplicate samples from three identical experiments, and error bars represent standard deviations. Statistically significant differences compared to Phi-1 are indicated by asterisks (*P < 0.01).

Finally, the contribution of *lefA* to intracellular growth in mammalian cells was examined in a human macrophage cell line, THP-1 cells. As shown in our previous study, *L. pneumophila* Phi-1 and Ofk308 displayed potent growth 24 h after infection. However, the *lefA* mutant failed to grow in THP-1. In addition, the *lefA* complemental strain showed restitution of potent growth at the same level as parental strain Ofk308 (Fig. 7). These results indicate that, although *lefA* was identified as an endosymbiosis modulation factor in protistan hosts in this study, it is also an essential factor for *Legionella* to grow intracellulary in THP-1 cells.

## Discussion

Protists are deeply involved in the life cycle of *L. pneumophila*^44^. Therefore, the development of protistan host models is greatly useful for investigating the mechanisms of *Legionella* infection and endosymbiosis, which occur in the natural environment. In addition, prior adaptation to intracellular growth within primitive eukaryotic hosts such as protists is thought to be required for *L. pneumophila* to gain the ability to infect humans and survive in macrophages^20^,^45^. In this study, we established a novel, endosymbiotic host model of *Legionella* symbiosis using *Paramecium* spp. The high mobility and high cell division rate of *Paramecium* in nature may be the major advantages for the spread of bacteria. In addition, all infection assays in our present work were performed at 25 °C, which is typical for *Paramecium* spp. culture conditions. Although this temperature is lower than that used in infection procedures reported in other protistan hosts^46^,^47^ or in macrophages, the cytotoxicity or endosymbiosis of *Legionella* strains in *Paramecium* hosts were clearly observed under these conditions. They reflect the natural environmental conditions under which *Legionella* survive, and reveal the true aspects of *Legionella* in the environment.

Two *L. pneumophila* strains isolated from the environment, Ofk308 and Bnt314, refused to establish endosymbiosis in 8 and 15 strains of *Paramecium*, including RB-1, respectively (Table 1). These *L. pneumophila* strains may have strict protistan host tropism or switching systems to select suitable hosts in the environment. Ofk308, in particular, may have employed systems correlating with *lefA* to survive and, as a result, to achieve some sort of advantage in the environment. It was reported that endosymbiont *Neochlamydia* in amoeba negatively affects subsequent *L. pneumophila* infection^2^. Thus, *lefA*-mediated switching systems may play a role in the elimination or avoidance of these inconvenient host cells.

Sodium/hydrogen antiporters are ubiquitous membrane proteins considered to be the major Na^+^ excretion system in bacteria^48^,^49^. Mutants lacking sodium/hydrogen antiporters are unable to grow in the presence of high concentrations of NaCl at pH 7^50^. However, their participation in virulence or intracellular growth is not well known in *Legionella*. The *lefA* mutant of *L. pneumophila* did not show growth depression in a medium containing a high concentration of NaCl, compared with parental strain Ofk308 (Supplementary Fig. 4). *L. pneumophila* Ofk308 is likely to have a number of genes encoding sodium/hydrogen antiporters or alternative transport systems that control Na^+^ excretion. Thus, it is possible that these genes substitute for *lefA*. On the other hand, the *lefA* mutant exhibited a complete deficiency of cytotoxicity toward *P. caudatum* RB-1 because of this single gene mutation. These results indicate that LefA has other unique functions contributing to cytotoxicity toward *P. caudatum* RB-1, aside from its role as a sodium/hydrogen antiporter. It was reported that another type of antiporter gene (*kefB*) from *Mycobacterium tuberculosis* arrests phagosomal maturation and acidification^51^. Ofk308 also inhibited acidification of LCVs in *P. caudatum* RB-1 and induced enlarged LCVs (Fig. 6). Ofk308 could modify the properties of LCVs depending on LefA, and make it possible for Ofk308 to grow, notably in RB-1. Thus, LefA may lead to the cytotoxicity toward RB-1. However, it was confirmed that other *Legionella* strains used in this study also have a homolog of *lefA*. We have investigated the expression levels of *lefA* in these *Legionella* strains, and observed up-regulation of *lefA* in Ofk308 due to uptake by RB-1 (Fig. 4b). These unidentified expression-regulating systems of *lefA*, or other factors associated with *lefA*, may be critical to exhibit the cytotoxicity.

Furthermore, although the Ofk308 strain showed potent growth, at the same level as that of *L. pneumophila* Phi-1, its *lefA* mutant failed to grow in THP-1 cells (Fig. 7). Our findings possibly provide another function of *lefA;* it is involved in intracellular growth in macrophage cells. It has been reported that several *Legionella* virulence factors identified in macrophages are also required for successful intracellular growth within protistan hosts such as amoebae^52^. It is well known that the Dot/Icm T4SS is critical for the intracellular growth of *L. pneumophila*^53^,^54^. The Dot/Icm secretion system injects effectors into the host cell, and enables *L. pneumophila* to adapt to intracellular life within both protistan and mammalian hosts^20^. Thus, we investigated the cytotoxicity of a *dotH*-deletion mutant of Ofk308 in *P. caudatum* RB-1. As a result, reduction of the cytotoxicity was not observed (Supplementary Fig. 5). These results suggest that the *lefA* may play a central role in the modulation of Ofk308 endosymbiosis in *P. caudatum* RB-1, independent of the Dot/Icm system.

In this study, we used 62 strains of 14 species in *Paramecium* as protistan hosts. There were differences in sensitivity among these strains to the cytotoxicity exerted by infection with Ofk308 and Bnt314 (Table 1). No definitive tendency of this sensitivity to cytotoxicity was found among these species. Two major types of taxonomy based on similarity of morphology have been reported in *Paramecium* spp. One method divides them into two groups: the aurelia group (*P. aurelia* species, *P. caudatum, P. multimicronucleatum*) and the bursaria group (*P. bursaria, P. putrinum, P. trichium, P. calkinsi*)^55^; and the other method divides them into three groups: the putrinum group (*P. putrinum, P. bursaria*), the woodruffi group (*P. woodruffi, P. calkinsi, P. polycarium, P. arcticum, P. preudotrichium*), and the aurelia group (*P. aurelia* species, *P. caudatum, P. jenningsi, P. africanum, P. multimicronucleatum, P. wichtermani*)^56^. We also evaluated whether any tendencies in the sensitivity to cytotoxicity exist according to these taxonomies. However, we could not find any clear correlations here, either. Studies of host-related factors are absolutely imperative in order to analyze the mechanisms of endosymbiosis by *L. pneumophila* in protistan hosts. Further examinations of the genetic backgrounds of these *Paramecium* strains may explain the differences in their sensitivity to cytotoxicity.

In conclusion, the results from our study suggest that *L. pneumophila* has a potential mechanism to control endosymbiosis in *Paramecium* in which the *lefA* gene is prominently involved. When the pathogenicity or infectious risks of pathogens that exist in environment, including *Legionella,* are investigated, it is important to know as much about their environmental phase before they infect humans. In that regard, the findings about the mechanism of endosymbiosis modulation shown in the *L. pneumophila-Paramecium* model contribute to the development of further research concerning environmental pathogens.

## Methods

### Bacterial strains

All bacterial strains and plasmids used in this work are listed in Table 2. *Legionella pneumophila* Philadelphia-1 (GTC_00296), Knoxville-1 (GTC_00745), and Togus-1 (GTC_00746) were obtained from National BioResouce Project (NBRP) of the Ministry of Education, Culture, Sports, Science and Technology, Japan ( http://www.nbrp.jp/). Five isolated environmental strains were reported in our previous study^21^,^57^. These *Legionella* strains were maintained as frozen glycerol stocks and cultured on either N-(2-acetamido)-2-aminoethanesulphonic acid -buffered charcoal yeast extract agar (BCYE) or in the same medium without agar and charcoal (AYE), at 37 °C. *E. coli* strains were cultured either in LB broth or on the LB containing 1.5% agar. If necessary, ampicillin (100 μg/mL), chloramphenicol (10 μg/mL), and kanamycin (30 μg/mL) were used. GFP expression in *Legionella* was induced by adding isopropyl-β-D-thiogalactopyranoside (IPTG) (1 μM) to AYE.

### Paramecium spp. strains

All *Paramecium* spp. used in this study were provided by Symbiosis Laboratory, Yamaguchi University with support, in part, by the NBRP. Culture and maintenance of *Paramecium* spp. were described previously^58^. In brief, the culture medium used for *Paramecium* was 2.5% (w/v) fresh lettuce juice in Dryl’s solution^59^, inoculated with a non-pathogenic strain of *Klebsiella pneumoniae* 1 day before use. The cultivation of *Paramecium* spp. was performed at 25 °C.

### Cytotoxicity measurement

Bacteria were added to each *Paramecium* spp. in 1.5 mL tubes at a multiplicity of infection (MOI) of 10, 100, 1000, and 10000, respectively. Killed bacteria were obtained by treatment with 4% paraformaldehyde for 60 min. After washing 3 times with PBS, these killed bacteria were added to RB-1 in a 1.5 mL tube at a substantial MOI of 1000. Overnight cultures of bacteria were centrifuged at 15 000 rpm for 5 min, and then the supernatants were filtered through a 0.45-μm filter. These culture supernatants were also added to RB-1 in a 1.5 mL tube. These tubes were incubated at 25 °C for 48 h. After incubation, the number of live *Paramecium* was counted by microscopy.

### Transposon mutagenesis

Random mini-Tn5 transposon mutagenesis was used to generate mutants of the *L. pneumophila* Ofk308 strain. The mini-Tn*5*-bearing plasmid pUTmini-Tn*5* Km (BioMedal S. L.) was introduced into the Ofk308 strain by electroporation with a Gene Pulser electroporator (Bio-Rad Laboratories) in a 10% glycerol solution at 2.5 kV/25 μF. The mutants were purified on agar plates containing kanamycin (30 μg/mL) and were screened for cytotoxicity toward RB-1. Chromosomal DNA of the mutant was extracted from an overnight culture using the DNeasy Blood & Tissue kit (QIAGEN). The identification of mini-Tn5 inserted region was performed by whole-genome sequencing of the mutant using paired-end sequencing with an Illumina MiSeq kit v3.

### Determination of bacterial load in RB-1

RB-1 was infected with GFP-expressing *Legionella* or *E. coli* at an MOI of 1000. After incubation at 25 °C, the RB-1 was washed using a Celltrics Fillter (mesh size, 10 μm) (Sysmex Partec GmbH) to remove the extracellular bacteria. Furthermore, to purge the *K. pneumonia* fed to the RB-1, samples were treated at 50 °C for 30 min. Colony forming units (CFU) were determined by serial dilution on BCYE containing chloramphenicol (10 μg/mL).

### Fluorescence microscopy

GFP- or AsRed-expressing bacteria were added to RB-1 and were then incubated at 25 °C for 30 min to 48 h. Samples were fixed with 4% paraformaldehyde in PBS for 10 min at room temperature. Subsequently, samples were washed twice with PBS. Fluorescent images were obtained using a FluoView FV100 confocal laser scanning microscope (Olympus). To observe the ELCV and LLCV, *P. caudatum* RB-1 was infected with *L. pneumophila* Phi-1 and TRDx was added to the medium simultaneously. And then extracellular bacteria and TRDx were washed out by filtration 30 min after infection. At this point, CBDx was added to the medium (Fig. 1b). TRDx, CBDx, and pHDx (Life Technologies) were added to RB-1 with bacterial infection or at the indicated times at concentrations of 50 μg/mL. LysoTracker (Life Technologies) was used after fixation for 30 min at a concentration of 50 nM. Concanamycin A (1 μM, Wako), a V-type proton pump inhibitor, was added to RB-1 5 min after infection. The number of these indicator-positive vacuoles was counted by microscopy, and shown as an average of 10 RB-1 cells.

### Quantitative real-time PCR

The RB-1 infection assay were performed as described above. The bacterial RNA were extracted using the TRIzol regent (Life Technologies). Total bacterial RNA were subjected to reverse transcription using the ReverTra Ace qPCR RT Master Mix with gDNA Remover (Toyobo) according to the manufacturer’s instructions. Quantitative assays were performed on each cDNA with the THUNDERBIRD SYBR qPCR Mix (Toyobo) using an Applied Biosystems StepOne Real-Time PCR System. Dissociation curve analysis was performed in order to verify product homogeneity. The 16S rRNA amplicon was used as an internal control in order to normalize all data. The relative expression levels of the genes of interest were calculated using the relative quantification method (ΔΔC_T_). The specific primers for each target were designed as listed below: 5′-GTAGGCAAGTGCAGCAAACA and 5′-TGCATTTACAGCCCCAGAAT for *lefA*, and 5′-TAAGGAGACTGCCGGTGACA and 5′-GGCCATTGTAGCACGTGTGT for 16S rRNA.

### THP-1 cells culture and infection assay

Cells from the human monocytic cell line THP-1 were grown in RPMI 1640 medium (Sigma–Aldrich), supplemented with 10% heat-inactivated FBS at 37 °C under an atmosphere containing 5% CO_2_. THP-1 cells were differentiated with 100 nM phorbol 12-myristate 13-acetate (Sigma–Aldrich) 48 h prior to use. Bacteria were added to a monolayer of THP-1 cells in 48-well tissue culture dishes at an MOI of 1. These plates were centrifuged for 10 min at 900 × *g* and incubated for 1 h at 37 °C. Extracellular bacteria were killed by gentamicin (30 μg/mL) treatment for 30 min. To measure the intracellular growth, the cells were incubated in fresh medium at 37 °C for the designated amount of time, washed three times with PBS, and then lysed with cold distilled water. CFU were determined by serial dilution on BCYE.

### Statistical analyses

Statistical analyses were performed using Student’s *t*-test. Statistically significant differences compared with the control are indicated by asterisks (*P < 0.01). Data are the averages of triplicate samples from three identical experiments, and the error bars represent standard deviations.

## Additional Information

**How to cite this article**: Watanabe, K. *et al*. Ciliate *Paramecium* is a natural reservoir of *Legionella pneumophila. Sci. Rep.* **6**, 24322; doi: 10.1038/srep24322 (2016).

## Supplementary Material

Supplementary Information

## Figures and Tables

**Table 1:** Cytotoxicity toward *Paramecium* spp. by infection with *Legionella*.

No	*Paramecium spp.*			*L. pneumophila*							
	NBRP ID	Species	Strain Name	Philadelphia	Knoxville	Togus	Ymg289	Twr292	Ymt294	Ofk308	Bnt314
1	PA040015A	*tetraurelia*	d4-2 (7.2b)	−	−	−	−	−	−	−	−
2	PT040011A	*tetraurelia*	51	−	−	−	−	−	−	−	++
3	PT040015A	*tetraurelia*	d4-2	−	−	−	−	−	−	−	−
4	PT041001A	*tetraurelia*	st110-1a	−	−	−	−	−	−	−	−
5	PT042002A	*tetraurelia*	st110-1b	−	−	−	−	−	−	−	−
6	PA060001A	*sexaurelia*	GSZ-3	−	−	−	−	−	−	−	−
7	PA080002A	*octaurelia*	131	−	−	−	−	−	−	−	+
8	PA090001A	*novaurelia*	Ep-17	−	−	−	−	−	−	−	−
9	PA120001A	*dodecaurelia*	251	−	−	−	−	−	−	+	+
10	PA130001A	*tredecaurelia*	321	−	−	−	−	−	−	−	+
11	PB000006A	*bursaria*	KNZ0902g	−	−	−	−	−	−	−	−
12	PB000014A	*bursaria*	HA1g	−	−	−	−	−	−	−	−
13	PB031001A	*bursaria*	HK4g	−	−	−	−	−	−	−	−
14	PB031002A	*bursaria*	KM2g	−	−	−	−	−	−	++	++
15	PB033003A	*bursaria*	HK1g	−	−	−	−	−	−	−	−
16	PB031010B	*bursaria*	Yad1g1N	−	−	−	−	−	−	−	−
17	PB032001A	*bursaria*	Dd1g	−	−	−	−	−	−	−	−
18	PB033001A	*bursaria*	CT39g	−	−	−	−	−	−	−	−
19	PB032002A	*bursaria*	YF1g	−	−	−	−	−	−	−	−
20	PB034003A	*bursaria*	STL3g	−	−	−	−	−	−	+	+
21	PB034004A	*bursaria*	HA1g	−	−	−	−	−	−	−	+
22	PC000001A	*caudatum*	BW1	−	−	−	−	−	−	−	−
23	PC000021A	*caudatum*	Fura1	−	−	−	−	−	−	−	−
24	PC000028A	*caudatum*	NDI1-12	−	−	−	−	−	−	−	−
25	PC000030A	*caudatum*	NSA1	−	−	−	−	−	−	+	++
26	PC000033A	*caudatum*	RC307	−	−	−	−	−	−	+	++
27	PC000035A	*caudatum*	SDIA1-3	−	−	−	−	−	−	−	+
28	PC000044A	*caudatum*	KOM1231	−	−	−	−	−	−	−	−
29	PC000050A	*caudatum*	Kr302	−	−	−	−	−	−	−	−
30	PC000052A	*caudatum*	YD-10	−	−	−	−	−	−	−	−
31	PC000053A	*caudatum*	YD-11	−	−	−	−	−	−	−	−
32	PC011011A	*caudatum*	Mmn64	−	−	−	−	−	−	−	−
33	PC121023A	*caudatum*	KNZ1209	−	−	−	−	−	−	−	−
34	PC012002A	*caudatum*	Myn92	−	−	−	−	−	−	−	−
35	PC012005A	*caudatum*	KNZ1207	−	−	−	−	−	−	−	−
36	PC032013A	*caudatum*	TAZ0460	−	−	−	−	−	−	+	+
37	PC032024A	*caudatum*	HC3	−	−	−	−	−	−	−	++
38	PC032004A	*caudatum*	TAZ0462	−	−	−	−	−	−	−	-
39	PC041002A	*caudatum*	N93-027	−	−	−	−	−	−	−	−
40	PC042001A	*caudatum*	RB-1	−	−	−	−	−	−	++	++
41	PC042003A	*caudatum*	Hot1	−	−	−	−	−	−	++	+
42	PC051001A	*caudatum*	YD-1	−	−	−	−	−	−	−	−
43	PC061001A	*caudatum*	Yhb2-2	−	−	−	−	−	−	−	−
44	PC122029A	*caudatum*	dKNZ1207x1209-1	−	−	−	−	−	−	−	−
45	PC121031A	*caudatum*	dKNZ1207x1209-3	−	−	−	−	−	−	−	−
46	PC122033A	*caudatum*	dKNZ1207x1209-5	−	−	−	−	−	−	−	−
47	PC121001A	*caudatum*	My43C3d	−	−	−	−	−	−	−	−
48	PC131001A	*caudatum*	Atk	−	−	−	−	−	−	−	−
49	PD000001A	*duboscqui*	PD1	−	−	−	−	−	−	−	−
50	PK000001A	*calkinsi*	GN5-3	−	−	−	−	−	−	−	−
51	PM000003A	multimicro*	M03c4	−	−	−	−	−	−	−	−
52	PM000006A	multimicro*	M09	−	−	−	−	−	−	−	−
53	PM000009A	multimicro*	TH103	−	−	−	−	−	−	−	−
54	PM000011A	multimicro*	YM8	−	−	−	−	−	−	−	−
55	PM000014A	multimicro*	YM-26	−	−	−	−	−	−	−	−
56	PM034001A	multimicro*	YM-25	−	−	−	−	−	−	−	−
57	PN000001A	*nephridiatum*	Rw-1	−	−	−	−	−	−	−	+
58	PO000001A	*polycaryum*	YnA (+)	−	−	−	−	−	−	−	−
59	PP000001A	*putrinum*	WS1	−	−	−	−	−	−	−	−
60	PP001001A	*putrinum*	OM1	−	−	−	−	−	−	−	−
61	PP002002A	*putrinum*	Sw2	−	−	−	−	−	−	−	−
62	PP004001A	*putrinum*	OM4	−	−	−	−	−	−	−	−

−; no cytotoxicity (80–100% of survival rate), +; low cytotoxicity (less than 50% of survival rate), ++; high cytotoxicity (less than 10% of survival rate). *multimicro; *multimicronucleatum*.

**Table 2:** Bacterial strains and plasmids used in this study.

Strain	Characteristics	Reference or Sourse
*L. pneumophila*		
Philadelphia-1	Isolated from human lung	GTC 00296 (ATCC 33216)
Knoxville-1	Isolated from human lung	GTC 00745 (ATCC 33153)
Togus-1	Isolated from human lung	GTC 00746 (ATCC 33154)
Ymg289	Isolated from environmental water	Tachibana *et al*.^21^
Twr292	Isolated from environmental water	Tachibana *et al*.^21^
Ymt294	Isolated from environmental water	Tachibana *et al*.^21^
Ofk308	Isolated from environmental water	Tachibana *et al*.^21^
Bnt314	Isolated from environmental water	Tachibana *et al*.^21^
*lefA*	mini-Tn5Km inserted into *lefA* gene of Ofk308	This work
*lefA*/*lefA*^+^	*lefA* carrying pMS8-*lefA*	This work
*E. coli*		
DH5α	*Φ80lacZ*Δ*M15,* Δ*(lacZYA-argF)U169, recA1, endA1, hsdR17, supE44,thi-1, gyrA96, relA1*	Takara
DH5α λpir	DH5α (λpir) *tet*::Mu *recA*	Takara
JM109	*recA1, endA1, gyrA96, thi-1, hsdR17, e14- (mcrA-), supE44, relA1,* Δ *(lac-proAB)*	Takara
Plasmids		
pAM239-GFP	pMMB-derived vector encording GFP	Watarai *et al*.^60^
pAcGFP1	pUC-derived vector encoding GFP	Clontech
pAsRed2	pUC19-derived vector encording AsRed2	Clontech
pUTmini-Tn5Km	pUT vector containing mini-Tn5 carring Km resistance gene	BioMedal
pMS8-*lefA*	pMS8 vector expressing *lefA*	This work
