# Supplementary material for: Ciliate Paramecium is a natural reservoir of Legionella pneumophila
Source: Sci Rep. 2016 Apr 15;6:24322. doi: 10.1038/srep24322 (PMC4832178; doi:10.1038/srep24322)
Supplement: Supplementary Information [file srep24322-s1.doc]

Supplementary information for

Ciliate *Paramecium* is a natural reservoir of *Legionella pneumophila*

Kenta Watanabe^1,2^, Ryo Nakao^3^, Masahiro Fujishima^4,5^, Masato Tachibana^6^, Takashi Shimizu^1,2^, and Masahisa Watarai^1,2^

^1^The United Graduate School of Veterinary Science, Yamaguchi University, Yamaguchi, Japan.

^2^Joint Faculty of Veterinary Medicine, Laboratory of Veterinary Public Health, Yamaguchi University, Yamaguchi, Japan.

^3^The Graduate School of Veterinary Medicine, Hokkaido University, Sapporo, Japan. ^4^Department of Environmental Science and Engineering, Graduate School of Science and Engineering, Yamaguchi University, Yamaguchi, Japan.

^5^National BioResource Project of Japan Agency for Medical Research and Development, Chiyoda-ku, Tokyo, Japan.

^6^Division of Biomedical Food Research, National Institute of Health Sciences, Setagaya-ku, Tokyo, Japan.

Correspondence author:

Dr. Masahisa Watarai,

The United Graduate School of Veterinary Science, Yamaguchi University,

1677-1 Yoshida, Yamaguchi 753-8515, Japan.

E-mail: watarai@yamaguchi-u.ac.jp


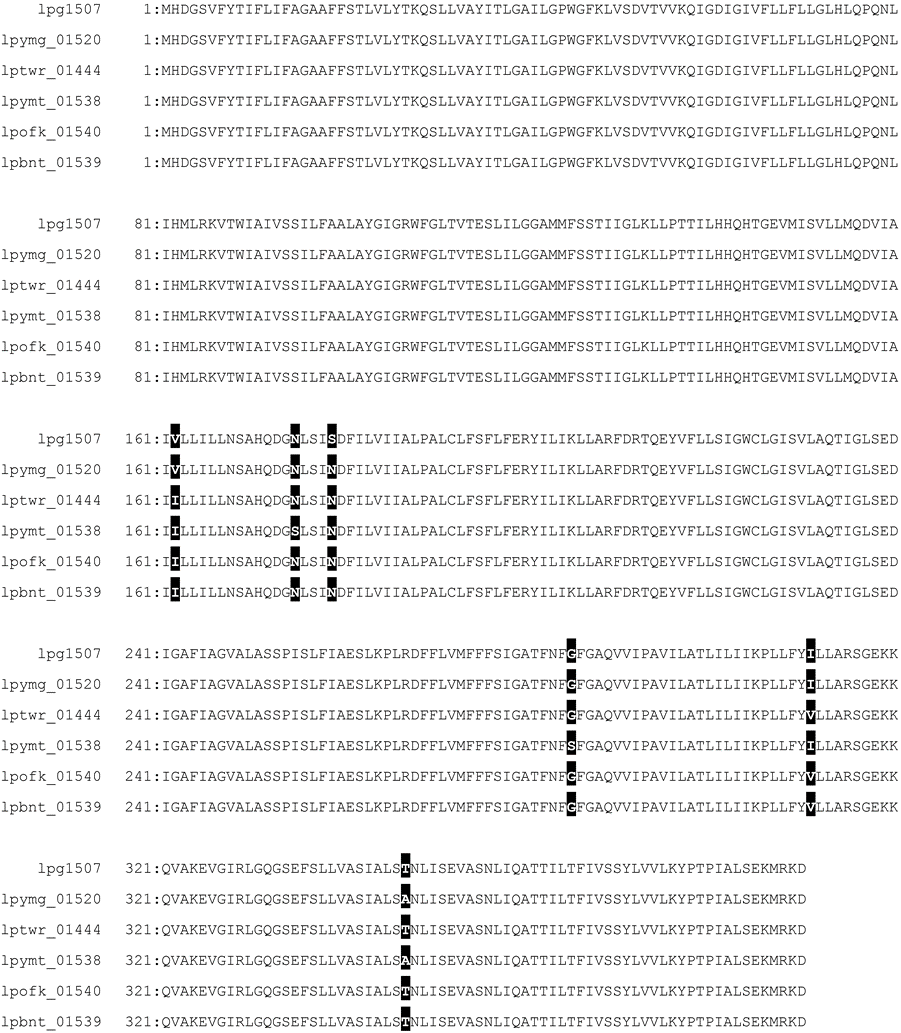


**Supplementary Figure 1. Alignment of LefA protein sequences.**

The different amino acid residues of LefA protein between 6 strains of *L. pneumophila* are shown in white characters on black background.


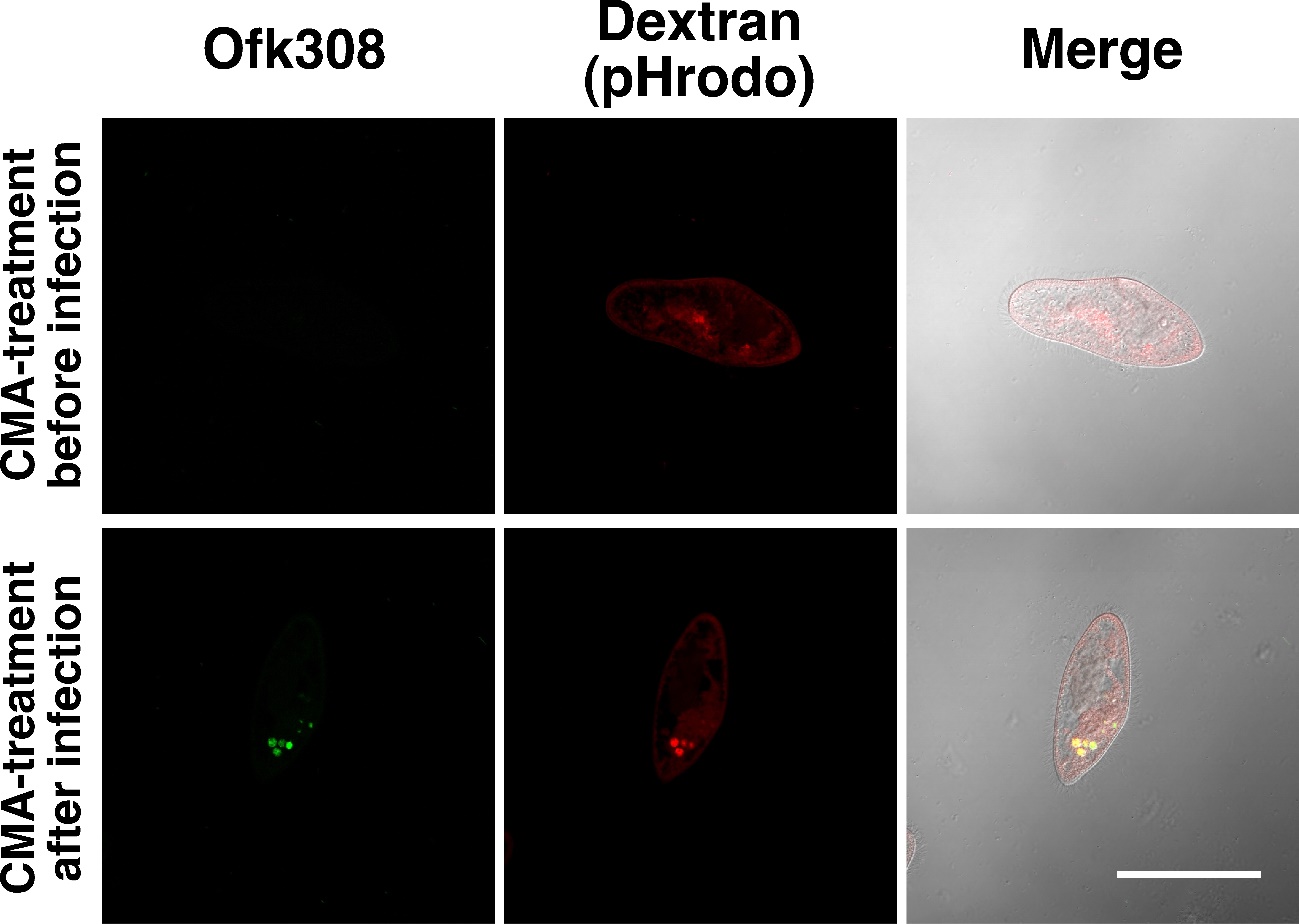


**Supplementary Figure 2. Pre-treatment of concanamycin A inhibits uptake of bacteria by RB-1.** RB-1 were infected with Ofk308 before or after treatment with CMA. Infected RB-1 were fixed and observed 30 min after infection. Scale bar represents 100 µm.


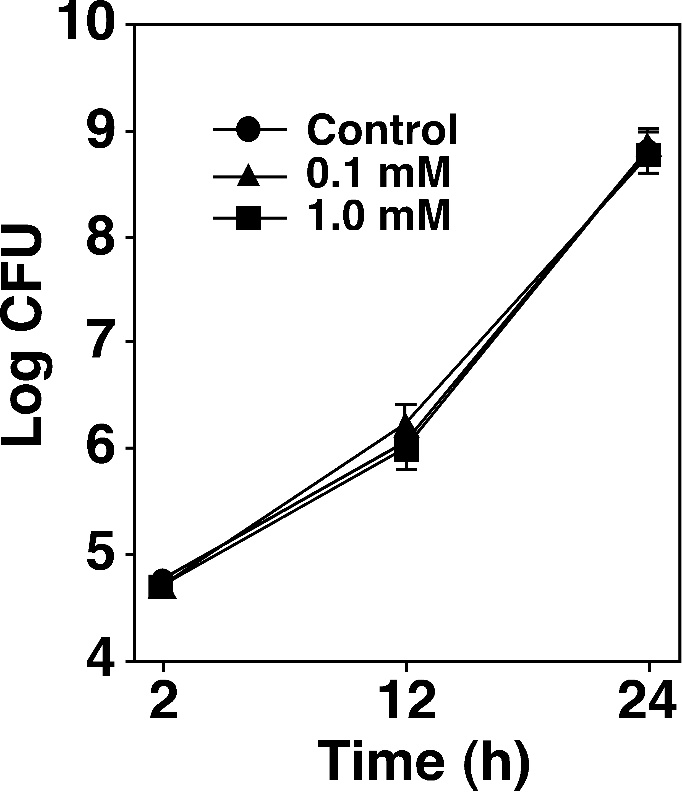


**Supplementary Figure 3. Treatment of concanamycin A has no effect for Ofk308 viability.** Ofk308 were cultured in AYE with CMA at the indicated concentrations. Data are averages of triplicate samples from three identical experiments, and error bars represent standard deviations.

**
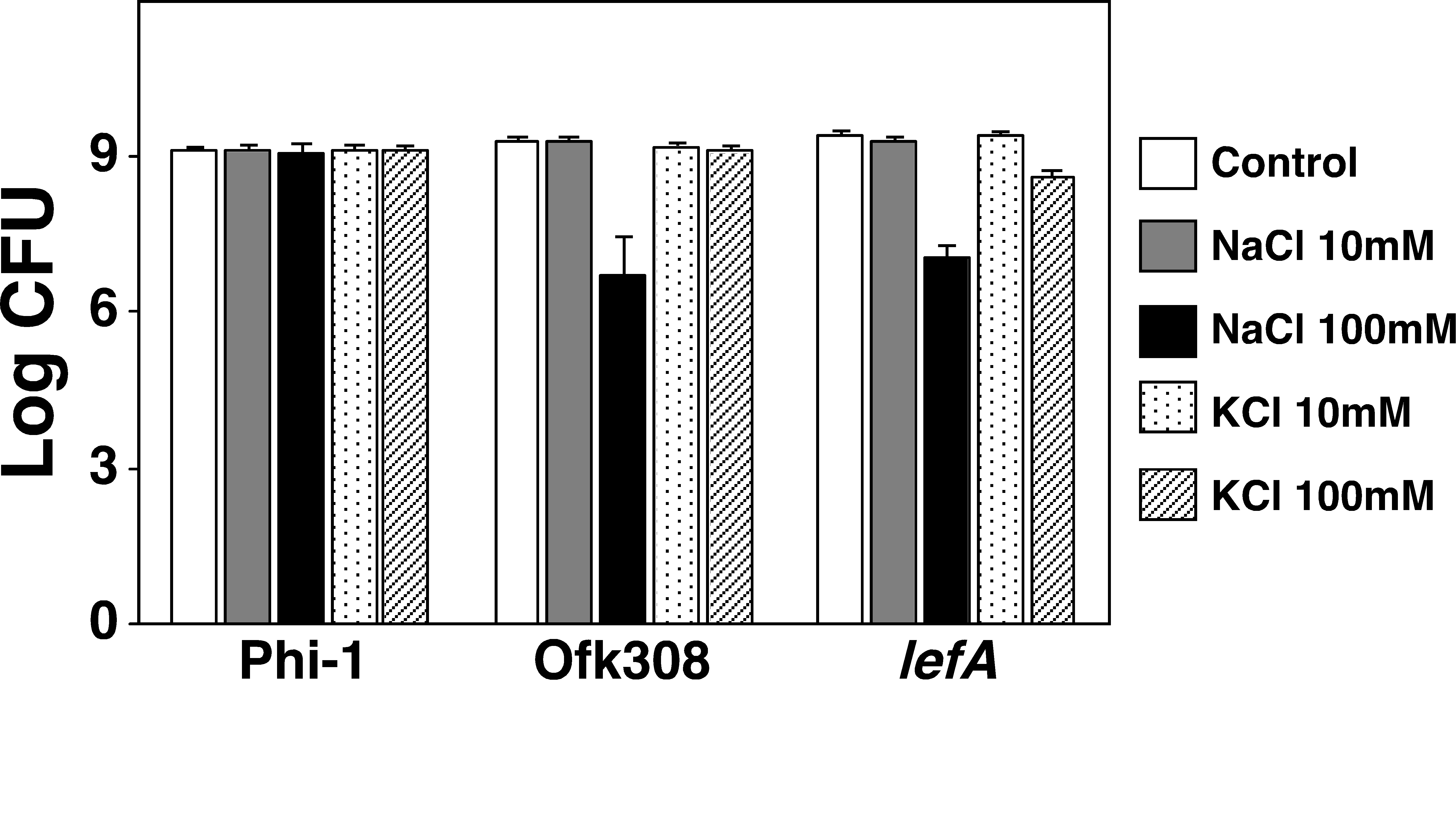
**

**Supplementary Figure 4. *lefA* mutant shows no sensitivity to high concentration of NaCl compared to Ofk308.** Phi-1, Ofk308, and *lefA* were cultured for 24 h in AYE containing NaCl or KCl at the concentration of 10 mM and 100 mM. CFU were determined by serial dilution on BCYE. Data are averages of triplicate samples from three identical experiments, and error bars represent standard deviations.


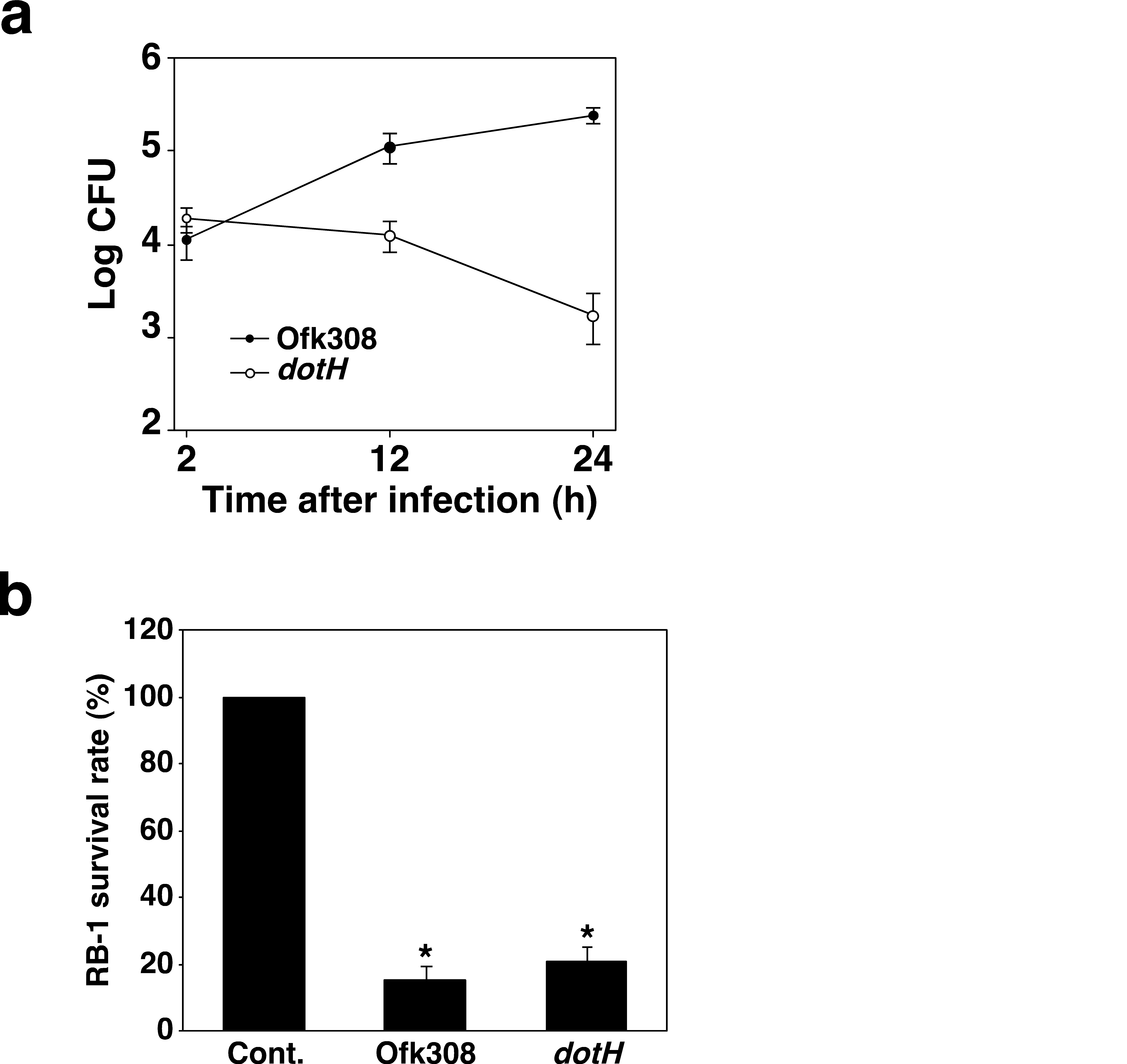


**Supplementary Figure 5.  *dotH* mutant fails to growth in THP-1 cells but have cytotoxicity toward RB-1.** (**a**) The THP-1 cells infected with *dotH* deletion mutant of Ofk308 (*dotH*) were cultured for 2, 12, and 24 h. Data are the averages of triplicate samples from three identical experiments, and the error bars represent standard deviations. (**b**) RB-1 were infected with Ofk308 and *dotH* at an MOI of 1000. Cont., no infection. Relative RB-1 survival rates were indicated with Cont. being defined as 100%. Data are averages of triplicate samples from three identical experiments, and error bars represent standard deviations. Statistically significant differences compared to Cont. are indicated by asterisks (*, P < 0.01).
